# Supplementary material for: Pulmonary Toxicity and Proteomic Analysis in Bronchoalveolar Lavage Fluids and Lungs of Rats Exposed to Copper Oxide Nanoparticles
Source: Int J Mol Sci. 2022 Oct 31;23(21):13265. doi: 10.3390/ijms232113265 (PMC9655042; doi:10.3390/ijms232113265)
Supplement: Supplementary file 1 [file ijms-23-13265-s001.zip › ijms-1948529-supplementary.pdf]

Pulmonary Toxicity and Proteomic Analysis in Bronchoalveolar Lavage Fluids and Lungs of Rats Exposed to Copper Oxide Nanoparticles

Jung-Taek Kwon[1,†], Yoonjin Kim[1,†], Seonyoung Choi[2], Byung-Il Yoon[3], Hyun-Sook Kim[4], Ilseob Shim[1], Donggeun Sul[2,*]

1 Environmental Health Research Department, National Institute of Environmental Research, Incheon, Republic of Korea,

2 Graduate School of Medicine, Korea University, Seoul 136-705, Republic of Korea

3 College of Veterinary Medicine and Institute of Veterinary Science, Kangwon National University, Chuncheon 24341, Republic of Korea,

4 Department of Biomedical Laboratory Science, College of Health Science, Cheongju University, Cheongju 28503, Republic of Korea

*Corresponding author: dsul@korea.ac.kr

†These authors contributed equally to this work

Journal: IJMS

Supplementary Information

A

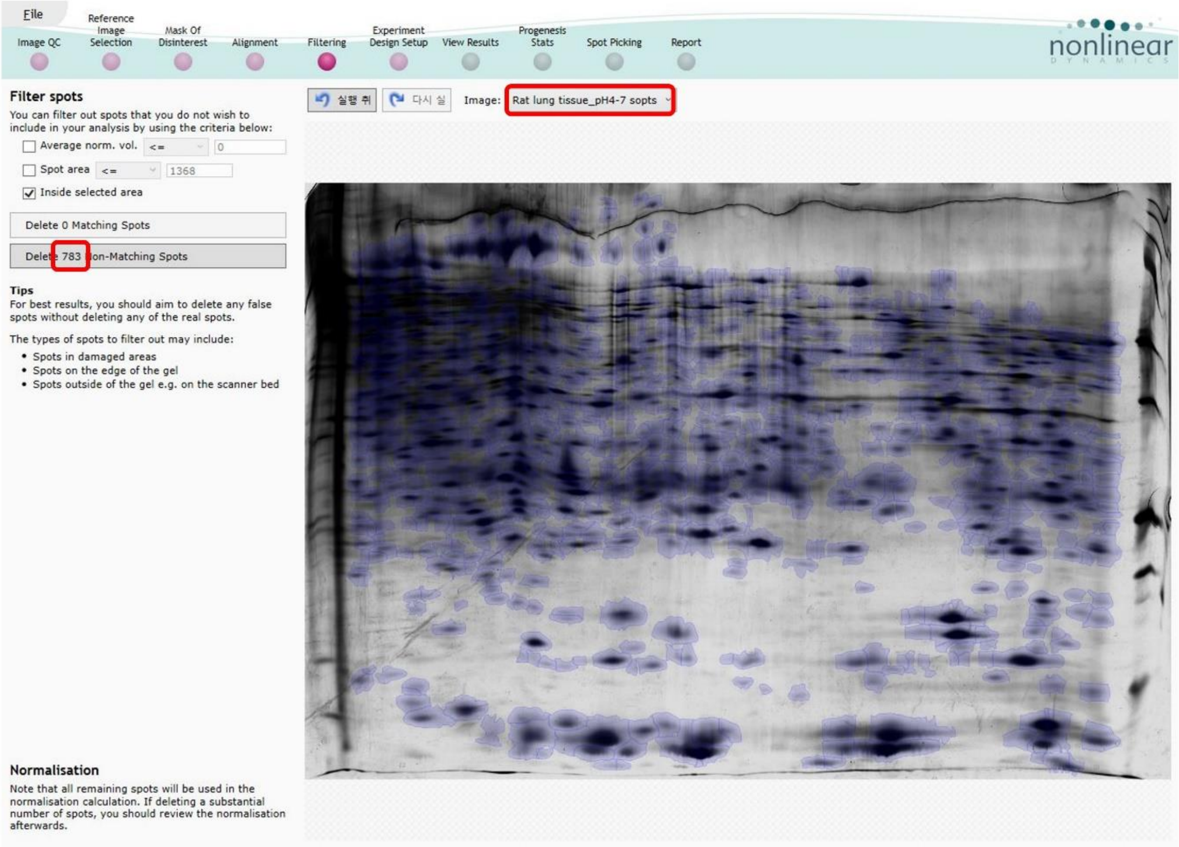
File
Image QC
Reference Image Selection
Mask Of Disinterest
Alignment
Filtering
Experiment Design Setup
View Results
Progenesis Stats
Spot Picking
Report
nonlinear
DYNAMICS
Filter spots
You can filter out spots that you do not wish to include in your analysis by using the criteria below:
Average norm. vol.
<=
0
Spot area
<=
1368
Inside selected area
Delete 0 Matching Spots
Delete 783 Non-Matching Spots
Tips
For best results, you should aim to delete any false spots without deleting any of the real spots.
The types of spots to filter out may include:
• Spots in damaged areas
• Spots on the edge of the gel
• Spots outside of the gel e.g. on the scanner bed
Normalisation
Note that all remaining spots will be used in the normalisation calculation. If deleting a substantial number of spots, you should review the normalisation afterwards.
실행 취
다시 실
Image:
Rat lung tissue_pH4-7 sopts

B

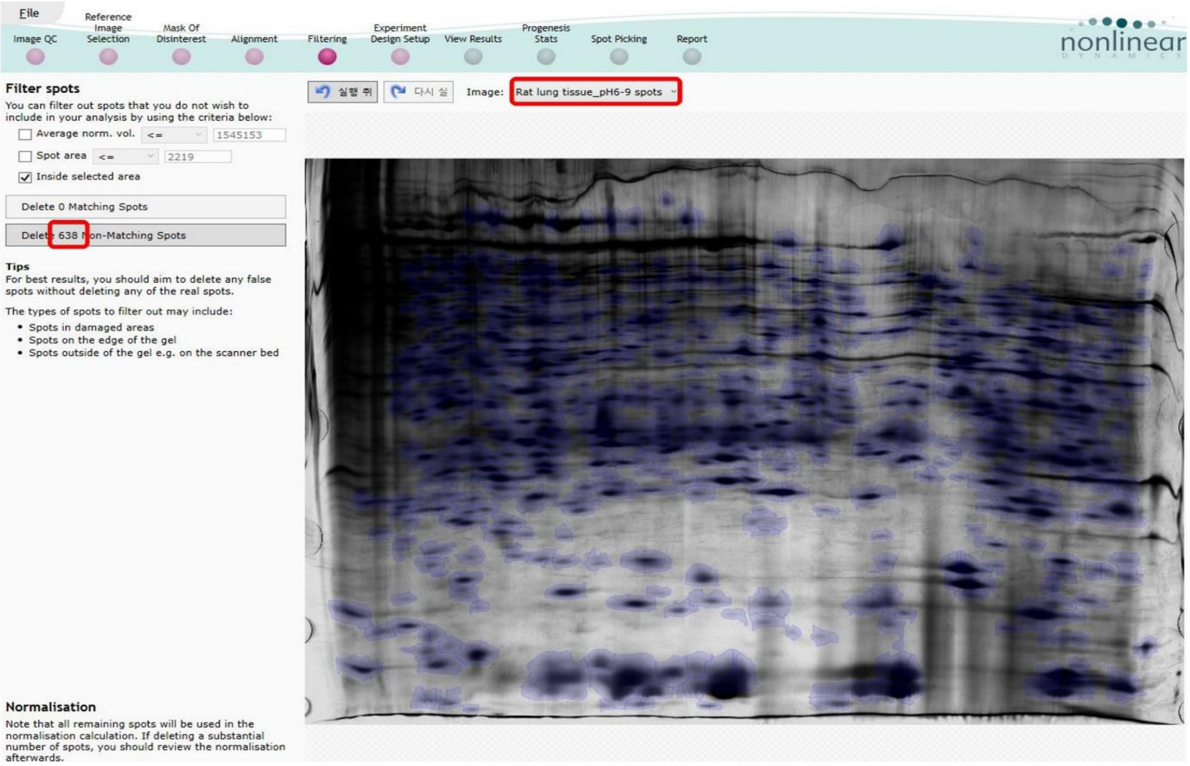
File
Image QC
Reference Image Selection
Mask Of Disinterest
Alignment
Filtering
Experiment Design Setup
View Results
Progenesis Stats
Spot Picking
Report
nonlinear
DYNAMICS
Filter spots
You can filter out spots that you do not wish to include in your analysis by using the criteria below:
Average norm. vol.
<=
1545153
Spot area
<=
2219
Inside selected area
Delete 0 Matching Spots
Delete 638 Non-Matching Spots
Tips
For best results, you should aim to delete any false spots without deleting any of the real spots.
The types of spots to filter out may include:
• Spots in damaged areas
• Spots on the edge of the gel
• Spots outside of the gel e.g. on the scanner bed
Normalisation
Note that all remaining spots will be used in the normalisation calculation. If deleting a substantial number of spots, you should review the normalisation afterwards.
실행 취
다시 실
Image:
Rat lung tissue_pH6-9 spots

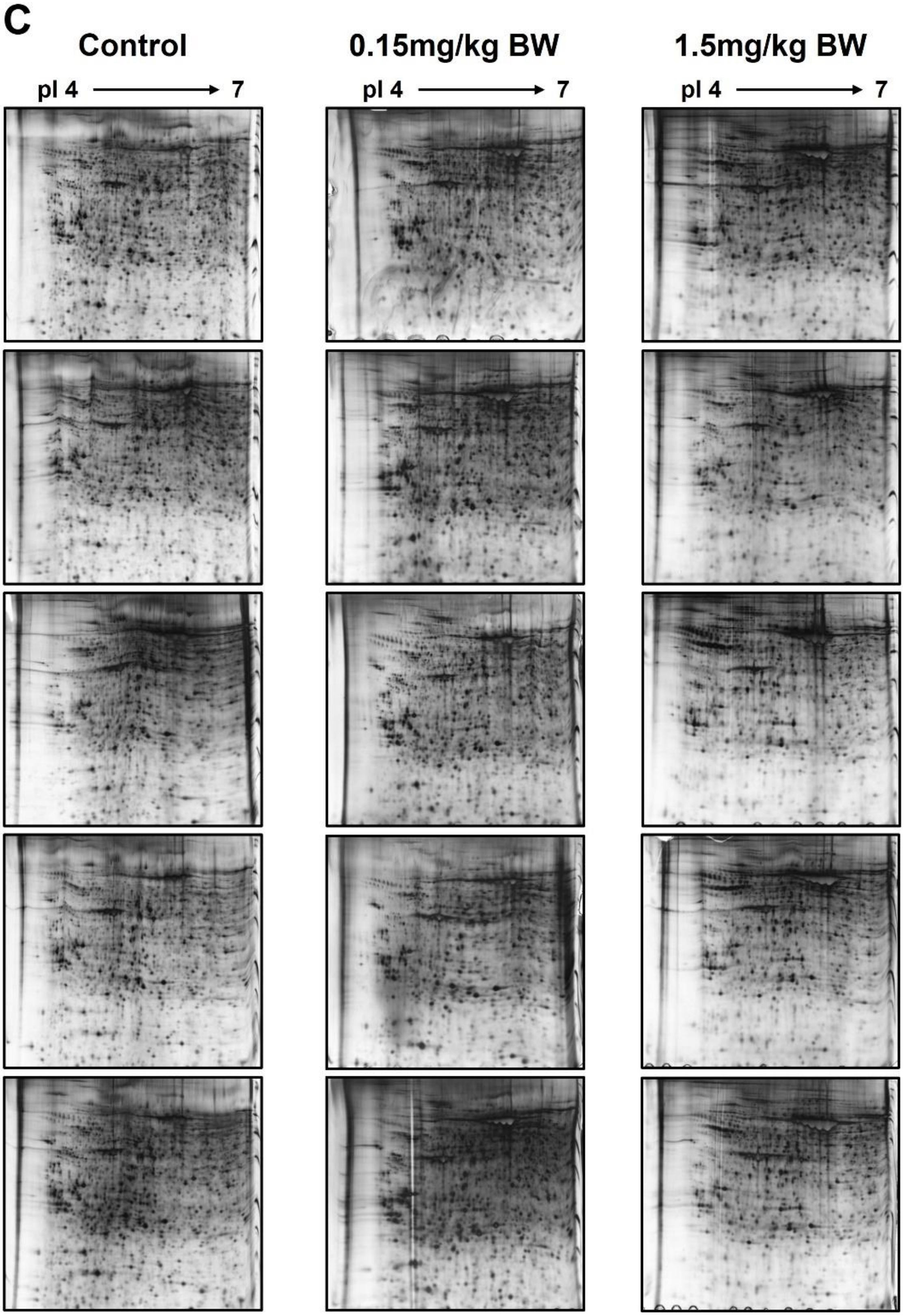
C
Control
0.15mg/kg BW
1.5mg/kg BW
pI 4
7
pI 4
7
pI 4
7

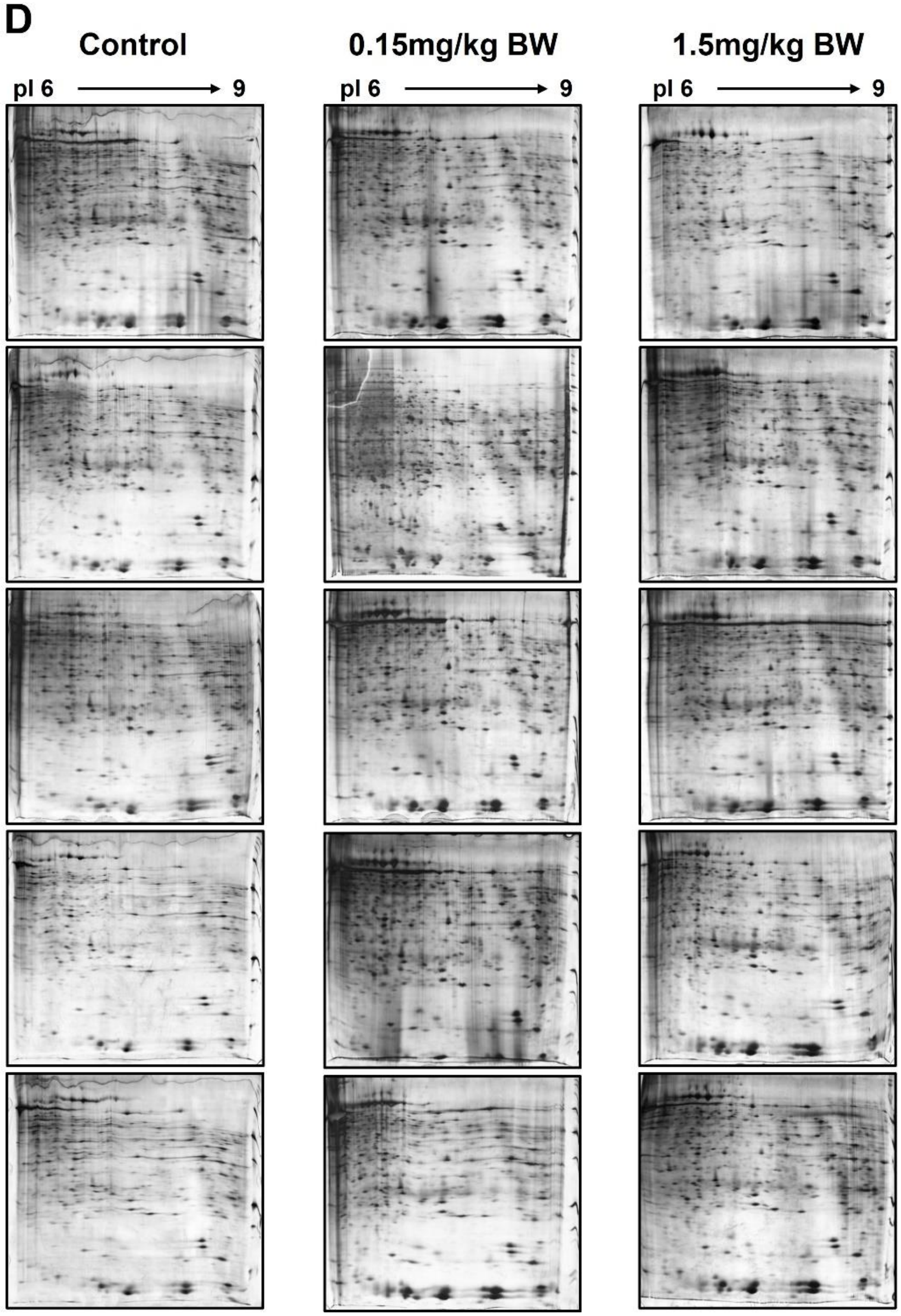

Figure S1. Supplementary image and original gel data: Gel image data. After separation on SDS-PAGE gels, the proteins were visualized by silver staining. The silver-stained gels were scanned using a 3600 × 4900 dpi instrument and the image files were transformed into a TIFF format with linear grayscale values. The computer analysis of the 2DE-image was carried out

using Progenesis Samespot image analysis software. Two different ranges of pI strips (4-7, and 6-9), showed 783 (**A**) and 638 (**B**) protein spots present in the gels, respectively. **Original gel data**, **(C)** Original gels (pI 4-7) and (**D)** Original gels (pI 6-9).

Table S1. Identification of proteins via UPLC-ESI-MS^E

Spot No.	Accession No.	Protein name	Sequence coverage (%)	Matched peptides	Theoretical peptides	mW (Da)	pI (pH)	Products	Products RMS Mass Error (ppm)	Products RMS RT Error (min)	Protein ID
1	P17425	Hydroxymethylglutaryl CoA synthase cytoplasmic	10.2	3	40	57397	5.48	28	33	0.0208	3100
2	F7FIH7	Protein LOC100909412	37	11	19	20904	5.54	60	23.29	0.0213	8626
3	Q6GX84	Fidgetin like protein 1	7.8	4	60	74150	6.61	29	37.15	0.0231	2479
4	M0RBF1	Complement C3	30.1	138	139	186205	6.02	1204	21.84	0.0229	9870
5	D3ZDK8	Uncharacterized protein	23.5	4	12	11016	4.55	28	33.29	0.0184	19936
6	P02091	Hemoglobin subunit beta 1	47.6	10	13	15969	8.25	196	23.11	0.0193	3033
7	F1LZT6	Protein Asap2 Fragment	6.6	6	75	103093	6.36	53	30.3	0.0247	26089
8	F1LWD5	Protein 1700056E22Rik	9.4	3	11	25323	10.14	35	22.6	0.0205	19533
9	M3ZCQ3	Protein LOC100910765	79.3	14	8	12132	6.61	84	18.24	0.0211	24625
10	Q3B8N7	TSC22 domain family protein 4	13.2	6	30	40016	7.41	40	39.47	0.022	6850
11	O35817	A kinase anchor protein 14	4.8	7	48	55721	10.19	40	27.83	0.0351	297
12	D3Z8I7	Protein Gstt3	19.5	8	17	23366	9.26	51	16.59	0.016	15387
13	P01946	Hemoglobin subunit alpha 1 2	48.6	19	10	15318	8.03	300	19.78	0.0178	3032
14	F1LQ93	Collagen alpha 1 IX chain	11.2	5	57	93768	9.84	42	33.38	0.0269	19366
15	P29457	Serpin H1	22.5	9	33	46488	9.19	67	35	0.0254	6338
16	Q07936	Annexin A2	45.1	19	31	38653	7.7	198	22.96	0.0188	406
17	P18420	Proteasome subunit alpha type 1	31.9	12	25	29498	6.15	77	29.13	0.0236	5508

Proteins were identified based on the search on the UniProt website (http://www.uniprot.org) from the Rattus norvegicus (Rat) database (28,063 entries).
